# Exploring the relationship between features calculated from contextual embeddings and EEG band power during sentence reading in Chinese

**DOI:** 10.3389/fnins.2025.1656519

**Published:** 2025-07-30

**Authors:** Yao Wang, Tiantian Xue, Xingyu Yang

**Affiliations:** ^1^Cognitive Science and Allied Health School, Beijing Language and Culture University, Beijing, China; ^2^Institute of Life and Health Sciences, Beijing Language and Culture University, Beijing, China; ^3^Key Laboratory of Language and Cognitive Science (Ministry of Education), Beijing Language and Culture University, Beijing, China

**Keywords:** contextual embeddings, vector-based neural coding, EEG, large language model, band power, neural oscillation

## Abstract

**Introduction:**

Contextual embeddings—a core component of large language models (LLMs) that generate dynamic vector representations capturing words’ semantic properties—have demonstrated structural similarities to brain activity patterns at the single-word level. This alignment supports the theoretical framework proposing vector-based neural coding for natural language processing in the brain, where linguistic units may be represented as context-sensitive vectors analogous to LLM-derived embeddings. Building on this framework, we hypothesize that cumulative distance metrics between contextual embeddings of adjacent linguistic units (words/Chinese characters) in sentence contexts may quantitatively reflect neural activation intensity during reading comprehension.

**Methods:**

Using large-scale EEG datasets collected during reading tasks, we systematically investigated the relationship between these computationally derived distance features and frequency-specific band power measures associated with neural activity.

**Results:**

In conclusion, gamma-band power exhibited associations with various NLP features in the ChineseEEG dataset, whereas no comparable gamma-specific effects were observed in the ZuCo1.0 dataset. Additionally, significant effects were found in other frequency bands for both datasets.

**Discussion:**

The mixed yet intriguing results invite a deeper discussion of the directional associations (positive/negative) observed in Gamma and other frequency bands, their cognitive implications, and the potential influence of textual characteristics on these findings. While observed effects may be somehow text- or dataset- dependent, our analyses revealed associations between various distance metrics and neural responses, consistent with predictions derived from the vector-based neural coding framework.

## 1 Introduction

The integration of electroencephalogram (EEG) techniques with large language models (LLMs) has recently gained significant attention in the field of psycholinguistics. Several studies have demonstrated a strong relationship between surprisal/entropy, derived from language models, and event-related potentials in EEG such as the N400 (Michaelov et al., 2023; Michaelov et al., 2024). Moreover, employing representational similarity analysis (RSA), recent research has revealed correspondences between the representational structures of LLMs and brain activity (Gillioz, 2024; Ren et al., 2024). In this trend, LLM-derived metrics not only predict electrophysiological responses but also establish systematic correspondences between artificial language representations and distributed cortical activation patterns.

Contextual and word embeddings - a core component of LLMs and Word2vec approaches, convert words into high-dimensional vector representations that encapsulate their semantic attributes. These embeddings facilitate semantic computations among words, exemplified by the well-known analogy “king – queen ≈ man – woman” (Levy and Goldberg, 2014). Unlike static word embeddings, contextual embeddings excel at capturing nuanced meanings depending on surrounding context, thus offering a more dynamic understanding of language. Recently, contextual and word embeddings have been leveraged in studies investigating how the brain processes language (Goldstein et al., 2024; Heilbron et al., 2022; Pereira et al., 2018). For example, Goldstein et al. (2024) provided compelling evidence for the use of this vector-based neural code at the single-word level. By analyzing the geometric patterns between brain embeddings (a continuous vectorial representation for each word derived from dense intracranial recordings in the inferior frontal gyrus) and contextual embeddings from LLMs like GPT-2, the researchers found significant correlations between these two sets of embeddings. This alignment suggests that words in the brain may be represented as dynamic vectors, similar to those generated by LLMs. These studies support a framework proposing a vector-based neural code for processing natural language in the brain.

The observed similarity between contextual embeddings and brain activity patterns at the single-word level gives rise to a compelling theoretical proposition: it is possible that the accumulated distance between contextual vectors of adjacent linguistic units (words or Chinese characters) within sentence structures could quantitatively correspond to neural activation intensity during language processing. Although our eyes may move back and forth during reading, our brain smooths out the discrete inputs so that we maintain a stable coherent view of the text sequentially (Rayner, 1998), for example, from left to right in English or from right to left in Arabic. In the context of vector-based neural coding, where contextual embeddings resemble brain embeddings, it’s natural to hypothesize that a larger cumulative distance or other potential features between adjacent word (or, Chinese character) vectors could indicate greater brain activity. In the current study, we aimed to investigate this possibility by linking such features to EEG band power which relates to brain activity.

To achieve this, various distance metrics between contextual embeddings should be considered. For example, Euclidean distance often reflects semantic similarity; words like “car” and “automobile” typically have a small Euclidean distance due to their closely related meanings. Cosine distance, on the other hand, measures directional similarity and is particularly useful in contexts where words like “doctor” and “hospital” appear in similar situations, leading to a smaller angle and thus a smaller cosine distance. Manhattan distance calculates the sum of absolute differences between corresponding components of vectors, while Chebyshev distance, which measures the maximum difference across any single dimension, may highlight the largest shift in meaning between two words. Each distance measure may provide unique insights that can be valuable depending on the specific linguistic or cognitive phenomenon under investigation. In addition to the distances between individual word vectors, the norms of sentence-level vectors, typically derived by averaging the vectors of each word, may also indicate the richness of the semantic information encoded within the sentence. Moreover, dimensionality reduction techniques can be employed to visualize the geometric properties of contextual embeddings. By projecting high-dimensional word vectors into a two-dimensional space, each word in a sentence is represented as a point in this 2D space. Researchers can then construct a convex hull around these points. The perimeter of the convex hull may suggest the breadth of semantic elements encompassed by the sentence, with a larger perimeter potentially indicating greater complexity and requiring more cognitive resources to process. Similarly, the area enclosed by the convex hull may represent the total semantic volume of the sentence.

To reflect the brain activity, EEG measures, including frequency-domain metrics like band power and time-domain metrics like N400 amplitude, are essential for understanding the dynamic shifts in cognitive load during sentence processing. Specifically, for sentence-level cognitive load, averaged EEG band power may be more informative. EEG band power can reflect different types of brain activity across frequency bands, with each band (or the coherence between bands) linked to specific brain functions and cognitive states. For instance, beta band power may relate to processes such as temporal integration and motor planning (Del Campo-Vera et al., 2020; van Helvert et al., 2021), while gamma band responses are often associated with attention and can serve as a quantitative marker of attentional processes (Durrheim et al., 2023; Hanslmayr et al., 2011; Klimesch, 1999; Liljeström et al., 2018). More relevant to the current study, previous research found that increases in gamma-band activity were closely related to semantic and phonological processes, thereby strongly linking the reading process to gamma activity (Vidal et al., 2012). Because the roles of other frequency bands in reading remain unclear, gamma-band power was the primary focus of the present study.

For the purposes of this study, a large-scale EEG dataset employing naturalistic reading tasks is essential. The Chinese sentence reading EEG dataset released by Mou et al. (2024) is particularly well-suited to our research objectives for two key reasons. First, each participant in this dataset read more than 10,000 sentences, greatly exceeding the scale of most comparable EEG corpora and providing high statistical power for examining neural correlates of language processing. Second, rather than rapid serial visual presentation (RSVP), their dataset employed whole-sentence presentation which is more suitable for the current study, enabling more naturalistic investigation of sentence comprehension. It should be noted, however, that the logographic nature of Chinese, including the absence of explicit word boundaries and character-based information encoding, may limit the generalizability of the findings. To ensure the robustness and cross-linguistic applicability of the current study, we additionally included the English ZuCo1.0 dataset (Hollenstein et al., 2018) in our analyses.

## 2 Materials and methods

All analysis scripts are available at OSF | Contextual embedding and EEG band power.

### 2.1 ChineseEEG dataset and EEG band power

A large-scale ChineseEEG dataset provided by Mou et al. (2024) was utilized for this study. In this dataset, each run was segmented into a series of units, with each unit containing no more than 10 Chinese characters. In their study, 10 participants were instructed to read two novels (the Chinese versions of *The Little Prince* and *Garnett Dream*), presented unit by unit (less than 10 characters). During this, both their EEG and eye-tracking data were collected. Mou et al. (2024) provided two preprocessed versions of the EEG data, each with different filter bands. For this study, we selected the version with the 0.5–80 Hz filter band and a sampling rate of 256 Hz (after down sampling). More details on the preprocessing can be found in their publication. Aside from their preprocessing, no additional preprocessing steps were taken to enhance the replicability of the entire analysis.

The processing of the preprocessed EEG data was done using Python-MNE (Gramfort et al., 2013). To ensure the accuracy of subsequent analyses, several electrodes (Electrodes near ears, forehead and back of head) are excluded from the dataset, these electrodes are either deemed irrelevant to the research focus or are prone to artifacts during data collection. After excluding these channels, the remaining 87 electrodes are used for further analysis.

The EEG data contains event markers that are crucial for segmenting the continuous data into discrete epochs. The focus of this study is on two specific event types: ROWS, Marks the beginning of an epoch and ROWE, Marks the end of an epoch. Epochs were extracted building on these marks.

The Welch method is applied to compute the PSD of the signal for each EEG channel. This method divides the data into overlapping segments, applies a window function (Hanning window), and then calculates the Fourier transform of each segment. The resulting spectrum is averaged over all segments to estimate the power at different frequencies. The parameters for the Welch method include the length of each segment used in the Fourier transform, which is dynamically set based on the duration of the epoch. For each epoch, the window length ranges from 0.5 s (minimum) to 1 s (maximum). The degree of overlap between consecutive segments is set to 50% (i.e., the number of overlapping samples between segments is half the segment length).

The power is calculated for several predefined frequency bands, each of which corresponds to specific cognitive states or processes: Delta: 1–4 Hz, theta: 4–8 Hz, alpha: 8–13 Hz, beta: 13–30 Hz, gamma: 30–50 Hz. For each frequency band, the indices of the frequencies that fall within the band’s range are identified from the computed PSD. Then, the power within the frequency band is calculated by integrating the PSD over the selected frequency range. This is done by summing the power values corresponding to the frequencies within the range of each band. Mathematically, this integration is done using the trapezoidal rule (np.trapz), which provides an approximation of the area under the curve of the PSD within the band. The power for each frequency band was calculated separately for each EEG channel. To obtain a summary measure per epoch, the average power across all channels was computed for each frequency band. This yielded the overall band power for each epoch in every frequency band.

### 2.2 Calculation of different features

The current analysis includes four cumulative distance metrics calculated from the contextual embeddings of each character: Total Euclidean Distance, Total Cosine Distance, Total Manhattan Distance, and Total Chebyshev Distance. These contextual embeddings were extracted from BERT-base-Chinese,^1^ where each Chinese character in a unit is transformed into a 768-dimensional vector. Cumulative distances for each unit were computed by summing the distances between adjacent contextual vectors within the unit.

Additionally, six unit-level norms were included in this study, consisting of three types of norms—Euclidean, Manhattan, and Chebyshev—extracted separately from two models: BERT-Chinese and Text2Vec.^2^ For the BERT-Chinese model, each character (e.g., 7 characters) in a unit was first transformed into contextual embeddings, resulting in an embedding matrix of size (7,768). The unit-level embedding was then obtained by averaging the first dimension of these embeddings, yielding a single vector of size (1, 768). For the Text2Vec model, the entire unit was directly transformed into a unit-level embedding.

In addition to the above features, three derived features from dimensionality reduction were included: the sums of neighboring point distances, the perimeter, and the area of the convex hull. The dimensionality of the character-level embeddings was reduced to two using Principal Component Analysis (PCA), projecting the embeddings onto a 2D plane. The perimeter and area of the convex hull formed by these points were computed, as well as the sum of the distances between neighboring points within the convex hull.

**Figure 1:**
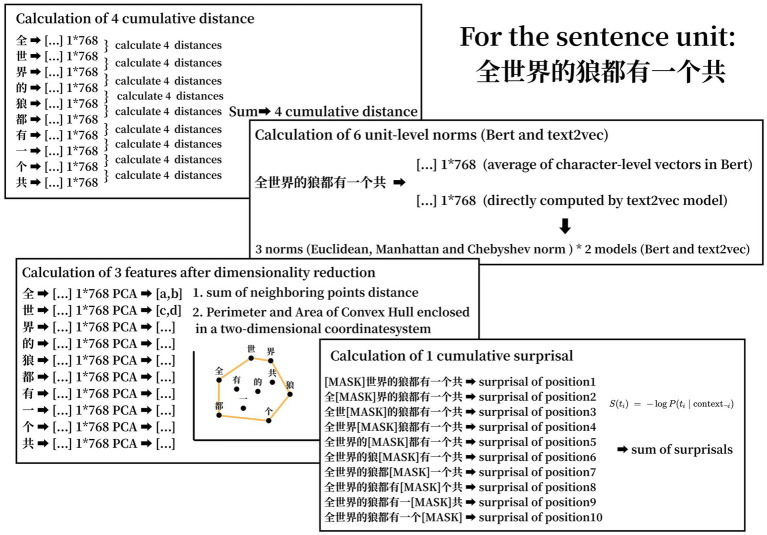
Example of how 14 features were computed.

For the calculation of cumulative surprisal, each sentence was first passed through BERT’s tokenizer to produce a sequence of subword tokens. Then, for each token position in turn, we created a “masked” input by replacing the original token with the special [MASK] token while leaving all other tokens unchanged. This masked sequence was fed into the BERT model to obtain the output probability distribution over the vocabulary at that masked position. We looked up the probability that BERT assigned to the original (unmasked) token, converted that probability into a token-level surprisal score by taking its negative log, and repeated this process for every token in the sentence. Finally, we summed all token-level surprisal scores to produce a single cumulative surprisal value for each sentence, providing a robust measure of overall unpredictability as estimated by the language model. An example of feature calculation was given in Figure 1.

### 2.3 Statistical analyses

To investigate the relationship between the 14 features and EEG band powers, we fitted Linear Mixed Effect Models (LMM) using the statsmodels package in Python (Seabold and Perktold, 2010). The 14 features were treated as fixed effects for each EEG band power, resulting in five separate LMMs, one for each frequency band. Each model included random intercepts for both participants and runs. Before analyses, the extreme values (a criteria of 2.5SD) were excluded from models. Importantly, since the dataset is informative enough, *The Little Prince* and *Garnett Dream* were analyzed separately to determine whether the results differed between these two sets of texts. In this analysis, no fixed effects were removed from the linear mixed models solely due to statistical insignificance. The primary aim was to explore potential factors that might explain the dependent variable, rather than focusing exclusively on significance levels. While the main threshold for significance was set at *α* = 0.05, factors with *p* < 0.1 were also reported.

### 2.4 ZuCo1.0 dataset and analyses

In the preprocessed ZuCo1.0 dataset (Hollenstein et al., 2018), 12 healthy, right-handed native English speakers read natural sentences while both EEG and eye movements were recorded. EEG was recorded with a 128-channel Geodesic HydroCel Sensor Net (Electrical Geodesics, Eugene, OR), sampled at 500 Hz with an analog band-pass of 0.1–100 Hz. Of those channels, 105 scalp electrodes and 9 EOG channels were retained for analysis; the remainder (face/neck) were discarded in their dataset and we use the 105 electrodes to calculate the averaged band power. After import into EEGLAB, data were high-pass filtered at 0.5 Hz and notch-filtered at 49–51 Hz, no further downsampling was applied—the data remained at 500 Hz throughout. For our study, we exclusively analyzed data from Task 2 (“Normal reading – Wikipedia”) of the ZuCo 1.0 corpus, as it most closely mirrors natural reading. In this task, participants passively read 300 sentences drawn from a Wikipedia relation-extraction corpus, selected from an initial pool of relation-containing candidates (The dataset was substantially smaller than ChineseEEG, which included over 10,000 sentences per participant, whereas the English data comprised only 300 sentences). One participant was excluded from our analyses due to a missing block of data.

In line with the Chinese EEG analysis, we used the English counterparts of the same embedding architectures—namely, BERT-base-uncased^3^ and Text2Vec.^4^ All feature extractions, band-power computations, and downstream analyses followed the same procedures as those used for the Chinese EEG data.

## 3 Results

### 3.1 The result of *the little prince* in ChineseEEG

**Figure 2:**
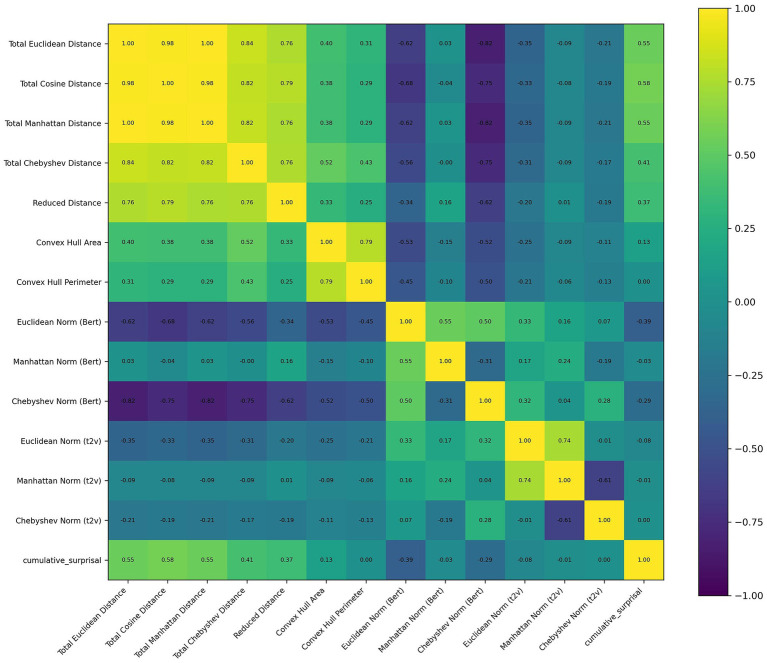
Correlation matrix of features used in the text of The Little Princes. Off-diagonal correlation coefficients reported as “1.00” in the heatmap are due to rounding.

The correlation matrix of these features was presented in Figure 2. No significant effects were found for the delta or theta bands. The only factor with a *p*-value below 0.1 was Total Cosine Distance (*β* = −2.251, *SE* = 1.157, *z* = −1.945, *p* = 0.052) in theta band, which did not reach the conventional significance threshold of *α* = 0.05.

However, for the alpha band, significant associations were identified with Total Cosine Distance (*β* = −1.512, *SE* = 0.405, *z* = −3.735, *p* < 0.001) and Euclidean Norm (Bert) (*β* = −1.034, *SE* = 0.334, *z* = −3.092, *p* = 0.002). For the beta band, only Total Cosine Distance (*β* = −1.303, *SE* = 0.682, *z* = −1.911, *p* = 0.056) and Euclidean Norm (Bert) (*β* = −0.958, *SE* = 0.563, *z* = −1.701, *p* = 0.089) were close to significance.

For the gamma band, significant effects were observed for Total Euclidean Distance (*β* = 12.419, *SE* = 6.028, *z* = 2.06, *p* = 0.039) and the Area of Convex Hull (*β* = 0.375, *SE* = 0.185, *z* = 2.02, *p* = 0.043). Although not reaching the conventional threshold of α = 0.05, Total Manhattan Distance (*β* = −0.509, *SE* = 0.262, *z* = −1.941, *p* = 0.052), Total Chebyshev Distance (*β* = −3.253, *SE* = 1.749, *z* = −1.86, *p* = 0.063), and Euclidean Norm Text2Vec (*β* = −42.595, *SE* = 23.275, *z* = −1.83, *p* = 0.067) were close to significance. A summary was shown in Table 1.

**Table 1:** Summary of results.

Features	β (Fixed Effects) in LMMs			
	The little princess		Garnett Dream	Combined
	Alpha	Gamma	Gamma	Gamma
Total Euclidean distance		12.419^*^		12.752^*^
Total Cosine distance	**−1.512** ^***^			
Total Manhattan distance				−0.557^*^
Euclidean Norm (Bert)	**−1.034** ^**^			
Manhattan Norm (Bert)				
Euclidean Norm (text2vec)			72.113^*^	49.902^*^
Manhattan Norm (text2vec)			−2.67^*^	
Convex Hull Area		0.375^*^		
Cumulative Surprisal			−1.054^*^	−1.03^*^

***p < 0.001; **p < 0.01; *p < 0.05, Boldface indicates results that remain significant after correction for multiple comparisons (*α* = 0.05/20 total tests), bands and features that did not reach significance are omitted for brevity.

### 3.2 The result of *Garnett dream* in ChineseEEG

**Figure 3:**
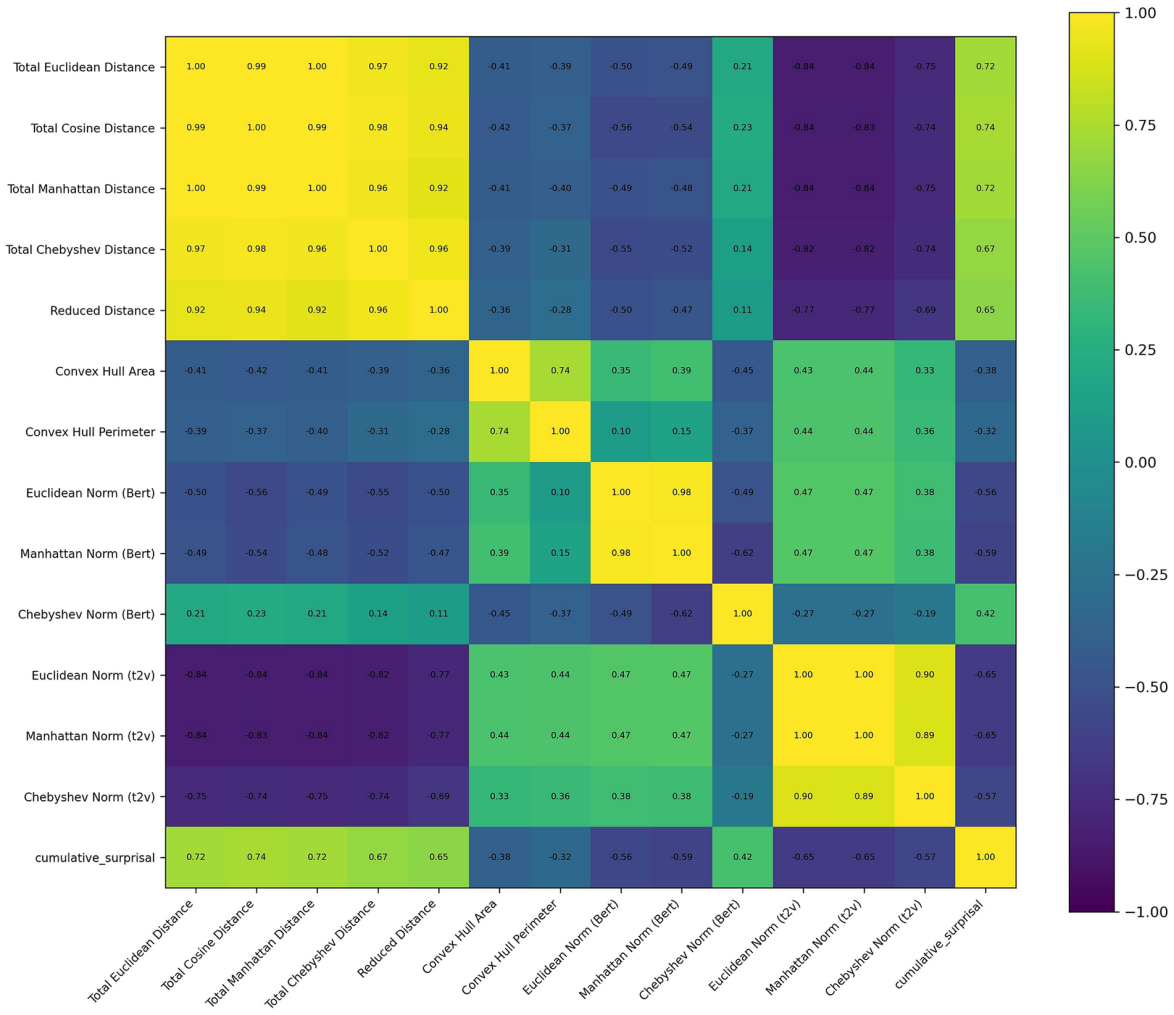
Correlation matrix of features used in the text of Garnett Dream. Off-diagonal correlation coefficients reported as “1.00” in the heatmap are due to rounding.

The correlation matrix of these features was presented in Figure 3. For the result of *Garnett Dream*, neither significant effect nor factors with *p* < 0.1 was observed in the model of delta, theta and beta. However, the Euclidean Norm Text2Vec (*β* = 72.113, *SE* = 28.211, *z* = 2.556, *p* = 0.011), Manhattan Norm Text2Vec (*β* = −2.67, *SE* = 1.107, *z* = −2.411, *p* = 0.016) and cumulative surprisal (*β* = −1.054, *SE* = 0.485, *z* = −2.172, *p* = 0.03) were significant in the model of gamma band. Also, Total Euclidean Distance (*β* = 13.613, *SE* = 7.558, *z* = 1.801, *p* = 0.072), Total Manhattan Distance (*β* = −0.591, *SE* = 0.33, *z* = −1.794, *p* = 0.073) and Chebyshev Norm Text2Vec (*β* = −14.551, *SE* = 8.669, *z* = −1.679, *p* = 0.093) were close to significance. In alpha band, the cumulative surprisal (*β* = 0.394, *SE* = 0.22, *z* = 1.794, *p* = 0.073) were close to significance.

Since the result of *Garnett Dream* was quite different from the result of *The Little Prince*, a subsequent combined analysis was done to obtain more information.

### 3.3 The combined results in ChineseEEG

The materials (Garnett Dream and The Little Prince) utilized in the experiment, along with the interaction between materials and each feature, were treated as fixed effects in the linear mixed models (LMMs). However, no significant interaction effects were observed in any of the models, and as a result, these interactions as well as material effect were excluded to simplify the model.

In terms of frequency bands, no significant effects were found for the delta, theta, alpha, and beta bands, although many fixed effects approached edge significance. For delta band, The Area of Convex Hull (*β* = 2.585, *SE* = 1.394, *z* = 1.855, *p* = 0.064) and Manhattan Norm (Bert) (*β* = −9.779, *SE* = 5.342, *z* = −1.83, *p* = 0.067) were close to significance. While for beta band, factors with *p* < 0.1 was the Area of Convex Hull (*β* = 0.139, *SE* = 0.075, *z* = 1.848, *p* = 0.065). For alpha band, the cumulative surprisal (*β* = 0.302, *SE* = 0.17, *z* = 1.777, *p* = 0.076) was close to significance.

However, in the gamma band, the Total Euclidean Distance (*β* = 12.752, *SE* = 6.465, *z* = 1.972, *p* = 0.049), Total Manhattan Distance (*β* = −0.557, *SE* = 0.282, *z* = −1.978, *p* = 0.048), Euclidean Norm Text2Vec (*β* = 49.902, *SE* = 24.248, *z* = 2.058, *p* = 0.04) and cumulative surprisal (*β* = −1.03, *SE* = 0.424, *z* = −2.432, *p* = 0.015) were significant. Several were close, including Manhattan Norm Text2Vec (*β* = −1.79, *SE* = 0.953, *z* = −1.878, *p* = 0.06) and Chebyshev Norm Text2Vec (*β* = −14.409, *SE* = 7.419, *z* = −1.942, *p* = 0.052).

### 3.4 The results in ZuCo1.0

**Figure 4:**
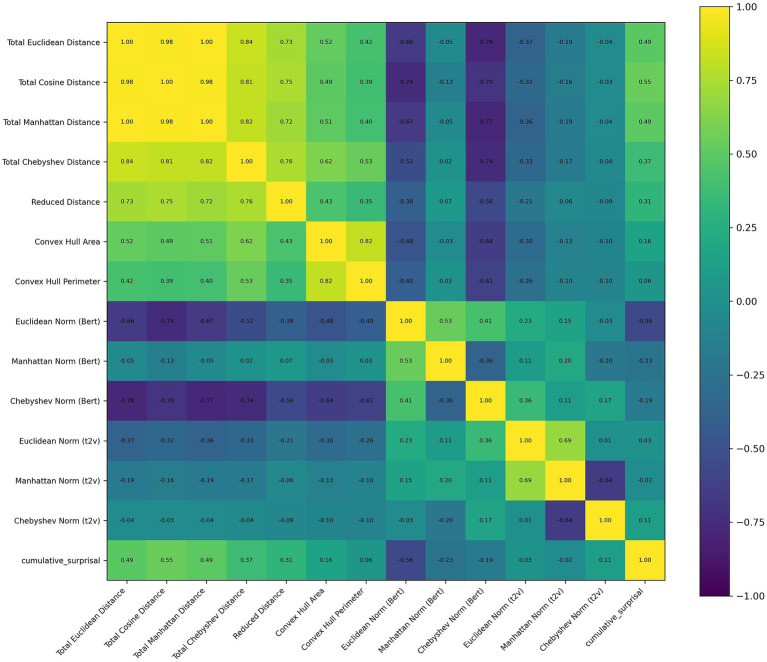
Correlation matrix of features used in the text of ZuCo1.0. Off-diagonal correlation coefficients reported as “1.00” in the heatmap are due to rounding.

The correlation matrix of these features was presented in Figure 4. No significant effect was observed in gamma band, which is different from ChineseEEG dataset. In the theta band, Chebyshev Norm (Bert) (*β* = 0.075, *SE* = 0.024, *z* = 3.126, *p* = 0.002) was significant. In the alpha band, Total Cosine Distance was significantly negatively associated with power (*β* = −0.144, *SE* = 0.036, *z* = −4.052, *p* < 0.001). Also in the alpha band, Convex Hull Perimeter showed a reliable negative effect (*β* = −0.028, *SE* = 0.008, *z* = −3.370, *p* = 0.001). In the beta band, Total Cosine Distance again emerged as a significant negative predictor (*β* = −0.065, *SE* = 0.025, *z* = −2.632, *p* = 0.008). A summary was presented in Table 2.

**Table 2:** Summary of results.

Band	Features	*β*	SE	*z*	*p*
Delta	Convex Hull Area	−0.002	0.001	−1.853	0.064
**Theta**	**Chebyshev Norm (BERT)**	**0.075**	**0.024**	**3.126**	**0.002**
**Alpha**	**Convex Hull Perimeter**	**−0.028**	**0.008**	**−3.37**	**0.001**
**Alpha**	**Total cosine distance**	**−0.144**	**0.036**	**−4.052**	**<0.001**
Beta	Manhattan Norm(Text2Vec)	0.022	0.013	1.669	0.095
Beta	Total cosine distance	−0.065	0.025	−2.632	0.008

A summary of result in ZuCo. Boldface indicates results that remain significant after correction for multiple comparisons (*α* = 0.05/20 total tests).

## 4 Discussion

### 4.1 Overview of findings

The current study mainly explored the relationship between features derived from contextual embeddings (as well as some other NLP features) and EEG band power during Chinese and English sentence reading, with a particular focus on gamma band activity. The mixed yet intriguing results invite a deeper discussion of the directional associations (positive/negative) observed in Gamma and other frequency bands, their cognitive implications, and the potential influence of textual characteristics on these findings. Because the ZuCo corpus is much smaller than ChineseEEG dataset, we give greater weight to the ChineseEEG results in our interpretations.

### 4.2 Gamma band: directionality and cognitive interpretation

Total Euclidean Distance (TED) exhibited a positive correlation with gamma power in *The Little Prince* and the combined analysis. TED quantifies the cumulative semantic divergence between adjacent characters in a sentence. A larger TED implies greater sequential shifts in meaning, which may demand heightened neural resources to integrate contextually distant concepts. This aligns with prior findings that Gamma power reflects attentional engagement and semantic binding during language comprehension (Penolazzi et al., 2009; Tierney et al., 2014). The weaker effects in *Garnett Dream* may stem from text-specific properties: *The Little Prince*, with its narrative simplicity, may emphasize incremental semantic shifts, whereas *Garnett Dream*’s potential complexity (e.g., abstract themes or dense syntax) might dilute the salience of TED as a global metric.

Area of Convex Hull (AoC) showed a positive correlation in *The Little Prince* but failed to reach significance in *Garnett Dream* or the combined analysis. The AoC represents the semantic “spread” of a sentence in a 2D PCA-reduced embedding space. Larger areas may suggest broader semantic diversity, which could necessitate greater neural synchronization to coordinate distributed representations. However, the reliance on PCA may introduce critical limitations. PCA prioritizes variance preservation but may obscure nonlinear semantic relationships, particularly in texts with intricate or abstract content (e.g., Garnett Dream). This could explain the inconsistency: simpler texts like *The Little Prince* may retain sufficient geometric structure in 2D to correlate with Gamma power, whereas complex texts lose discriminative power through PCA.

Cumulative surprisal exhibited a negative correlation with gamma power in Garnett Dream and the combined analysis, while Euclidean Norm (text2vec) exhibited a positive correlation with gamma power in the analysis. These opposing directions suggest that cognitive load is multidimensional, with different embedding-based features engaging distinct neural processes within the gamma frequency band.

### 4.3 Other frequency bands: marginal associations and theoretical implications

While gamma band dominated the results, weaker signals in other bands warrant discussion (though differences remain in different texts and datasets).

In both *The Little Prince* and ZuCo, Total Cosine Distance (TCD) showed significant negative correlations with Alpha power. Alpha oscillations are classically associated with cortical inhibition and reduced neural excitability (Clayton et al., 2018; Klimesch, 1999). The observed negative correlation suggests that sentences requiring less semantic reorientation (lower TCD, indicating smoother directional transitions between embeddings) may engage stronger alpha suppression. This suppression could reflect a disinhibition process, where reduced alpha power facilitates neural network activation for semantic integration (Hanslmayr et al., 2011). Also, in Zuco, TCD exhibited a marginal negative correlation with Beta power. The marginal effect and the absence of the effect in other texts limits interpretability, even if beta oscillations are implicated in higher-order linguistic functions, including syntactic maintenance and semantic memory retrieval (Lewis et al., 2016; Weiss and Mueller, 2012).

In all datasets, no effects reached significance in the delta band and only a single theta-band effect was detected, a pattern that aligns with the view that delta and theta oscillations predominantly support low-level sensory processing and memory encoding rather than higher-order linguistic functions (Basar and Duzgun, 2016; Haam et al., 2023; Rudoler et al., 2023).

### 4.4 Differences caused by datasets and languages

Although gamma-band power showed robust associations with different NLP features in the ChineseEEG dataset, no comparable effects emerged in the ZuCo1.0 data. This discrepancy may reflect the stark difference in corpus size—300 sentences per participant in ZuCo1.0 versus over 10,000 in ChineseEEG—leading to reduced power for detecting gamma-band relationships.

Besides the corpus size, another important factor is the different tokenization strategy used by LLMs for Chinese and English. In BERT-Chinese and related models, tokens almost invariably correspond to single Chinese characters, reflecting the logographic nature of the writing system and the absence of explicit word boundaries. In contrast, English LLMs such as BERT-base-uncased typically operate at the word or subword level, with most tokens representing entire words or common morphemes. As a result, the distance metrics computed from contextual embeddings in Chinese are based on transitions between characters, whereas in English, they are calculated primarily over word-level units.

This discrepancy in tokenization has important implications for the interpretation of embedding-derived features. In Chinese, cumulative distance metrics index fine-grained semantic transitions at the character level, potentially capturing local integration processes during sentence comprehension. By contrast, in English, these metrics aggregate over broader linguistic units, corresponding to coarser semantic or syntactic transitions. Consequently, the neural correlates of such metrics may reflect different aspects of language processing across the two languages, thereby complicating direct cross-linguistic comparisons of EEG-embedding relationships. Future research may benefit from harmonizing tokenization schemes or adopting alternative linguistic units (e.g., word-level embeddings for both languages) to facilitate more direct comparisons of semantic distance metrics and their neural correlates.

Beyond these technical differences, inherent language traits may also play a crucial role. Native English and Chinese speakers employ distinct reading strategies as a function of their respective language systems. Chinese script is logographic, with each character generally mapping to a morpheme and often a monosyllabic word, whereas English is alphabetic, with more transparent grapheme-to-phoneme correspondence and multi-letter word units. This distinction leads Chinese reading to rely more heavily on holistic visuospatial processing and morphemic/semantic integration, whereas English reading emphasizes phonological decoding (Li et al., 2022). These processing differences may underlie the more robust gamma-band effects observed in Chinese data: the greater visual and semantic complexity of Chinese characters could impose increased demands on distributed neural synchronization, particularly when semantic divergence or unpredictability (as indexed by embedding distances or surprisal) is high. Together, these factors highlight the need for future cross-linguistic work to systematically control for both technical and linguistic variables in order to clarify the universality and language-specificity of neural correlates of contextual embedding features.

## 5 Limitations

The limited sample size and text- and dataset- specific effects necessitates for replication with larger and more diverse cohorts. Also, while embedding-derived features offer rich insights, their cognitive interpretations remain indirect, canonical frequency bands support multiple, sometimes overlapping, cognitive operations, and that sentence-level embedding distances and norms represent relatively coarse summaries of the underlying processing. Furthermore, the focus on Chinese characters (a logographic system) limits generalizability; cross-linguistic studies are needed with extrapolation with alphabetic languages. Current study may also benefit from combing reading models such as E-Z reader. Comparative studies are needed to validate the universality of vector-based neural coding. The current study did not apply corrections for multiple comparisons, and the fitting of 20 linear mixed models (LMMs) may have reduced the reliability of the findings. Another limitation of the present analysis is the inclusion of highly correlated predictors (e.g., Total Euclidean Distance and Total Manhattan Distance, r = 0.99917) in the same model. While this approach allows for direct comparison of different distance metrics, it also introduces multicollinearity, which can inflate the standard errors of the coefficients and result in instability of significance estimates. Consequently, the interpretation of individual effects for these predictors should be made with caution, and the results primarily reflect the shared variance captured by these correlated measures.

Future studies could empirically validate embedding-derived metrics as objective indices of sentence difficulty and integrate them into stimulus design workflows in psycholinguistics. Building on these neural–linguistic associations, embedding-derived metrics such as cumulative surprisal or embedding-distance norms can be adopted as quantitative proxies for sentence difficulty when crafting psycholinguistic materials. Rather than relying solely on intuitive judgments, researchers could sample sentences at graded surprisal levels (e.g., low, medium, high) or along continuous ranges of Euclidean or cosine distance, ensuring that each condition imposes a predictable cognitive load.

## 6 Conclusion

This study offers preliminary evidence that features from contextual embeddings (as well as some other NLP features), particularly those capturing semantic divergence and geometric complexity, correlate with gamma band power during sentence reading. Future research should expand on these findings by integrating multimodal neural data and advanced NLP metrics to refine our understanding of the brain’s vector-based language encoding.

## Data Availability

The original contributions presented in the study are included in the article/supplementary material, further inquiries can be directed to the corresponding author/s.

## Footnotes

^1^google-bert/bert-base-chinese · Hugging Face

^2^shibing624/Text2Vec-base-chinese · Hugging Face

^3^google-bert/bert-base-uncased · Hugging Face

^4^shibing624/text2vec-base-multilingual · Hugging Face
